# Supplementary material for: Trends and determinants of caesarean section in South Asian countries: Bangladesh, Nepal, and Pakistan
Source: PLoS One. 2024 Dec 5;19(12):e0311082. doi: 10.1371/journal.pone.0311082 (PMC11620693; doi:10.1371/journal.pone.0311082)
Supplement: S1 Appendix — This file consists of Table A1: Percentages of delivery by C-section in urban-rural places of residence in Bangladesh, Nepal, and Pakistan at different DHS rounds. Table A2: Percentages of C-section delivery by administrative division in Bangladesh, Nepal, and Pakistan at different DHS rounds. Figure A1: Trends of birth at hospitals for three South Asian Countries: Bangladesh, Nepal, and Pakistan. Figure A2: Trends of C-sections in the three South Asian Countries: Bangladesh, Nepal, and Pakistan. (DOCX) [file pone.0311082.s001.docx]

# Appendix

Table A1: Percentages of delivery by C-section in urban-rural places of residence in Bangladesh, Nepal, and Pakistan at different DHS rounds

| Country | Survey year | Total births | C-section | |
| --- | --- | --- | --- | --- |
|  |  |  | Urban  % | Rural  % |
| Bangladesh | 2004 | 6987 | 10.6 (9.0-12.2) | 1.7 (1.3-2.2) |
|  | 2007 | 6055 | 15.9 (13.9-18.0) | 5.4 (4.4-6.6) |
|  | 2011 | 8777 | 25.8 (23.8-27.7) | 10.7 (9.6-12) |
|  | 2014 | 4626 | 40.0 (37.2-42.7) | 18.7 (16.5-21.1) |
|  | 2017-18 | 5331 | 43.8 (41.3- 46.4) | 28.7 (26.4-31.2) |
| Nepal | 2001 | 6977 | 4.8 (2.8- 6.8) | 0.6 (0.4-0.7) |
|  | 2006 | 5545 | 8.4 (6.4-10.5) | 1.9 (1.5-2.2) |
|  | 2011 | 5391 | 15.3 (12.2-18.5) | 3.5 (3.0-4.0) |
|  | 2016 | 5060 | 11.7 (10.5-12.9) | 5.9 (4.9-6.8) |
| Pakistan | 1990 | 6373 | 5.8 (4.8-6.8) | 1.4 (1.0-1.7) |
|  | 2006 | 9110 | 13.0 (11.7-14.2) | 4.9 (4.4-5.5) |
|  | 2012 | 11955 | 23.6 (22.2-25) | 10.2 (9.5-10.8) |
|  | 2017 | 10482 | 32.3 (30.7-33.8) | 17.7 (16.8-18.5) |

Note: % are weighted to account for survey design.

Table A2: Percentages of C-section delivery by administrative division in Bangladesh, Nepal, and Pakistan at different DHS rounds

| Country | Survey Year | Administrative Division | | | | | | | |
| --- | --- | --- | --- | --- | --- | --- | --- | --- | --- |
|  |  | Barisal | Chittagong | Dhaka | Khulna | Rajshahi | Sylhet |  |  |
| Bangladesh | 2004 | 2.5 (1-4) | 2.5 (1.7-3.3) | 5.2 (4.3-6.1) | 4.5 (3-6.1) | 2.1 (1.4-2.8) | 2.6 (1.3-3.9) |  |  |
|  | 2007 | 3.8 (1.9-5.8) | 6.5 (5.1-7.8) | 10.1 (8.7-11.4) | 9.7 (7.3-12.1) | 6.4 (5.1-7.8) | 4.3 (2.6-6) |  |  |
|  | 2011 | 9.8 (7.1-12.4) | 11.5 (10.1-12.9) | 16.7 (15.3-18.1) | 22.4 (19.5-25.3) | 11.8 (10.4-13.2) | 11.1 (8.8-13.5) |  |  |
|  | 2014 | 18.5 (13.8-23.1) | 19.5 (17-21.9) | 31 (28.8-33.3) | 34.4 (29.5-39.2) | 20.7 (18.1-23.3) | 12 (9-15.1) |  |  |
|  | 2017-18 | 24.5 (19.7-29.4) | 26.2 (23.6-28.7) | 38.6 (36.3-40.8) | 42.7 (38.3-47.1) | 31.9 (29.3-34.6) | 22.6 (18.6-26.6) |  |  |
| Nepal |  | Province 1 | Province 2 | Province 3 | Province 4 | Province 5 | Province 6 | Province 7 |  |
|  | 2001 | 1 (0.5-1.5) | 1.2 (0.8-1.7) | 0.8 (0.3-1.3) | 0.1 (-0.1-0.3) | 0.3 (-0.1-0.7) | Nil | Nil |  |
|  | 2006 | 1.9 (1.1-2.7) | 4.2 (3.3-5.1) | 3.1 (2.1-4.2) | 1.3 (0.5-2.2) | 0.8 (0.2-1.5) | Nil | Nil |  |
|  | 2011 | 1.4 (0.3-2.6) | 3.7 (2.9-4.5) | 5.8 (4.9-6.6) | Nil | Nil | Nil | Nil |  |
|  | 2016 | 12.7 (10.4-15) | 5 (3.9-6.2) | 17.4 (14.8-20) | 16.7 (12.9-20.4) | 6.4 (4.8-8) | 2.2 (0.6-3.8) | 3.1 (1.4-4.7) |  |
| Pakistan |  | Punjab | Sindh | KPK | Balochistan | GB | ICT | AJK | Fatah |
|  | 1990 | 3.1 (2.6-3.7) | 2.6 (1.8-3.5) | 1.8 (0.9-2.7) | 0.4 (-0.4-1.1) | Nil | Nil | Nil | Nil |
|  | 2006 | 9.2 (8.4-10) | 6.5 (5.5-7.5) | 3 (2-3.9) | 1.5 (0.3-2.7) | Nil | Nil | Nil | Nil |
|  | 2012 | 17 (16.1-17.9) | 15.4 (14.1-16.8) | 4.6 (3.6-5.6) | 1.5 (0.5-2.4) | 3.3 (-0.6-7.1) | 26.6 (13.7-39.6) | Nil | Nil |
|  | 2017 | 29.2 (28-30.4) | 22.9 (21.2-24.6) | 7.8 (6.5-9.1) | 4.1 (2.5-5.8) | Nil | 29.3 (19-39.7) | Nil | 2.7 (0.7-4.8) |

Note: % are weighted to account for survey design. Rangpur division of Bangladesh has been included into the Rajshahi division, because for the first three survey it was with the Rajshahi.


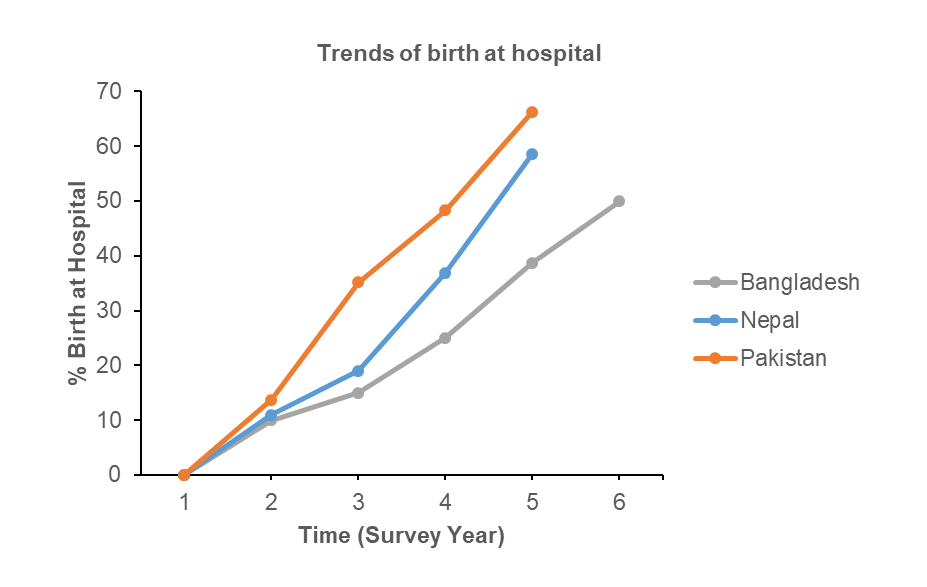


Figure A1: Trends of birth at hospitals for three South Asian Countries: Bangladesh, Nepal, and Pakistan.


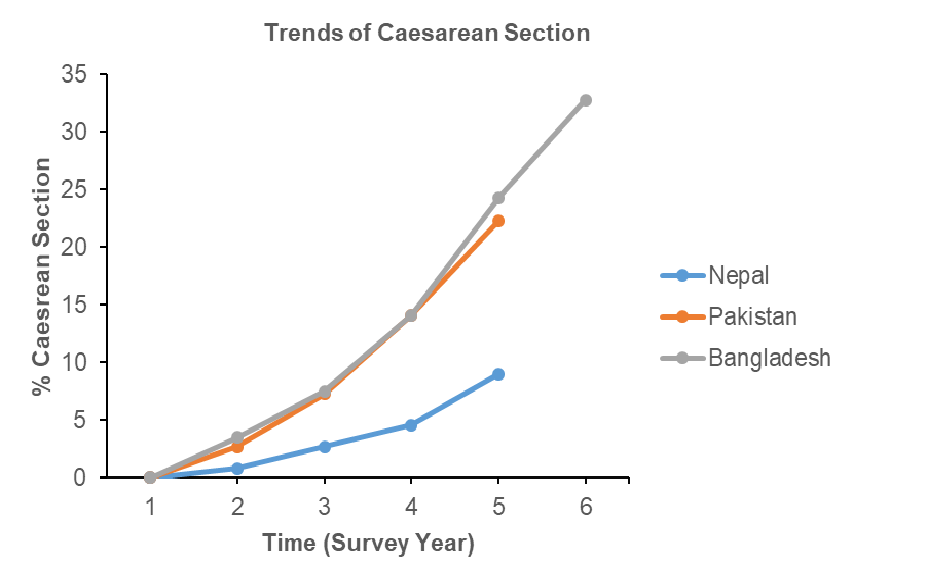


Figure A2: Trends of C-sections in the three South Asian Countries: Bangladesh, Nepal, and Pakistan.
