# Supplementary material for: Trends and determinants of caesarean section in South Asian countries: Bangladesh, Nepal, and Pakistan
Source: PLoS One. 2024 Dec 5;19(12):e0311082. doi: 10.1371/journal.pone.0311082 (PMC11620693; doi:10.1371/journal.pone.0311082)
Supplement: Attachment — Submitted filename: Response to Reviewers.docx [file pone.0311082.s002.docx]

| **Reviewer 1** | **Author responses** |
| --- | --- |
| 1. The author (s) must give proper justification to compare these three countries; saying these are neighbouring countries is not a sufficient argument. The justification/explanation must be from a healthcare/MCH perspective. Moreover, the Authors argued that the C-section rate in Nepal is still under the WHO recommended range, so there is no significance to comparing Nepal with Bangladesh and Pakistan. | Thanks a lot for your suggestion. We have tried to justify reasons of selecting Bangladesh, Nepal and Pakistan in our study according to your suggestions. |
| 2. The study's findings are similar to the DHS report; the author should go one-step further to estimate the prevalence of caesarean section in rural/urban areas or public/private areas in these countries. Furthermore, it has been recommended that, author should estimate state / region-wise prevalence of caesarean section and construct GIS map for better visualization. It will also help to strengthen the current study and policymakers to identify the area with high C-section. | Thank you very much for your comments and suggestions. We have included further analysis in the appendix where we showed prevalence of C-section by place of residence and administrative regions. |
| 3. The author must include the variable "type of hospital/facility" in which Caesarean Section delivery have been performed in this analysis. It helps to understand why CS rises, especially because of private facilities across the counties. If these countries have any MCH/delivery subsidies scheme then need to explain in the discussion section. | Thanks a lot for your scientifically important comment. We will do it in our future paper. |
| 4. Is Table 3 a pooled analysis of the association of c-sections with socio-demographic factors? If yes, need to write in the methodology section with justification and, if no, then mention the year in the table title. Similarly, for the odds ratio table 4, Is it pooled dataset? | Thank you very much for your comment. We did pooled analysis in table 3 and table 4. We included this information in the methods section according to your suggestions. Thanks again for your precious comments. |
| **Reviewer 2** |  |
| The authors address an important public health issue in the countries discussed. However, the manuscript does an unsatisfactory job of proving the premise, rationale and justification of the study goals. The manuscript does not seem well-written, (in many cases) overinterpret its results, and requires significant work to be of publishable quality. | Thank you very much for your comments. We have tried our best to comply with your valuable comments in the following section. We appreciate your valuable input and believe scientific importance will be increased because of fulfilment of your comments. |
| **Major Points** |  |
| 1.The manuscript provides considerably outdated, misappropriated and (in some cases completely irrelevant) references in the Background section. The authors were also unable to provide references where they are required even though the total number of references used is almost 60. | We appreciate your comments. We have updated citations and imported references in the required and appropriate places of the manuscript. |
| 2.The study tends to over-interpret its results and does a very unsatisfactory job of describing the premise of the public health issue in the Background and conducting a literature review. The authors’ repeated use of similar analysis of the same data set from past years as the justification for some of their claims is problematic. | Thanks for your comments. So far, we know there were no study on the similar topic for the same sets of data for the same three countries. |
| 3.The authors’ rationale behind selecting the 3 countries was “Since Bangladesh, Nepal and Pakistan is neighbouring countries, so this study will enable to compare trends of C-section and its determinants”, which does not necessarily shed light behind exclusion of other South Asian countries (for example India shares border with all 3 countries and shares many of the same sociodemographic components but was not included in the analysis). Do the three countries selected have similar health infrastructure to facilitate direct comparisons? If so, in what ways are they similar? Do the socio-cultural dissimilarities prohibit/encourage elective C-sections for one country over the other? | We do accept your comments warmly. We have enclosed proper justification of selecting the three countries (Bangladesh, Nepal and Pakistan) according to the maternal and child health perspectives. |
| 4.In the Abstract the authors note that: variables which were significant in chi-square test included as explanatory variables. (There is no statistical basis for performing statistical analyses in this way). However, in the methods section it is stated that model selection was performed using “backward elimination” (which again is a flawed approach as model selection should be based on sociodemographic domain and theoretical considerations and not just statistical significance). | Thank you very much for your comments. We highly appreciate your comments and suggestions. For your kind, information exposures were selected based on sociodemographic and theoretical considerations by doing literature reviews. However, those variables were included in the model showing significant association with c-section in the chi-squared test. It’s statistically valid, please have a look at the attached papers: <https://doi.org/10.1371/journal.pone.0257782>  <https://doi.org/10.3390/ijerph18084131>  <https://doi.org/10.1016/j.ntt.2004.01.002>  Also, it is statistically a valid methods of model selection either by forward selection, backward selection or stepwise selection:  <https://doi.org/10.1007/s12665-021-09998-5>  <https://doi.org/10.1002/cem.1360>  <https://doi.org/10.1080/03610910701723625> |
| **Abstract** |  |
| 1. “There is a paucity of studies exploring trends and determinants of C-section in the three South-Asian countries: Bangladesh, Nepal and Pakistan”. This claim is not necessarily true as there have been studies on this issue in these countries. For example, the following papers are similar studies in Bangladesh and Nepal: doi: 10.1136/bmjopen-2014-005982,[https://d­oi.org/10.1038/s41598-021-96337-0](https://doi.org/10.1038/s41598-021-96337-0); also using same DHS (BD and Nepal) data from past years: [doi.org/10.1371/journal.pone.0202879](http://doi.org/10.1371/journal.pone.0202879), <https://doi.org/10.1186/s12884-020-03453-2>, DOI: 10.1155/2021/8888267, and exact same data from Pakistan (<https://doi.org/10.1186/s12884-020-03457-y>). | Thanks a lot for your comments and suggestions. This (,[https://d­oi.org/10.1038/s41598-021-96337-0](https://doi.org/10.1038/s41598-021-96337-0)) study dealt with 2019 MICS data. doi: 10.1136/bmjopen-2014-005982 it is based on four cluster randomized control trial of participants from 2005 2012 in Bangladesh, India and Nepal. This paper ([doi.org/10.1371/journal.pone.0202879](http://doi.org/10.1371/journal.pone.0202879)) based on 2014 BDHS data, <https://doi.org/10.1186/s12884-020-03453-2> it is based on DHS data but only for Nepal, this (DOI: 10.1155/2021/8888267) is also only for Nepal and finally, this (<https://doi.org/10.1186/s12884-020-03457-y>) is for Pakistan which used DHS data. But, we mean there is no evidence of comparison of three South-Asian countries by pooling the DHS data. However, we warmly accepted your suggestions and tried to cite those papers in our manuscripts. |
| 2. The manuscript does use the most recent DHS data, which is indeed correct. But these datasets have been released fairly recently. | Heartfelt gratitude for your valued comments. Yes, we used all recent and past DHS data to present concrete evidence. |
| 3. Although C-section is a lifesaving delivery method at emergency, unnecessary use of it is *a financial* burden for the family. | Thanks again for your criticism. We believe the upgradation of the manuscript to comply with your comments and suggestions will improve its scientific value. |
| 4. “By reducing the influential factors respective authorities should minimize the over reliance on C-section during the childbirth” – Not sure how the authors could prescribe to reduce demographic attributes such as: mothers age, place of residence, years of education, partner’s years of education, wealth status, birth order, number of antenatal care visits, and body mass index. | We are grateful for your comments. Yes, actually the sentence will be ‘by modification of influential factors (body mass index, birth order, and wealth). We have made the correction, and would like to thank you. |
| 5. “Moreover, more mass media campaign should be initiated to create awareness among the stakeholders about the negative impacts of unnecessary C-sections” – The study did not consider media as a variable in the analyses and found any association. This is clearly extrapolating the results far outside the scope of the manuscript. | Many thanks for raising the issue. We are completely agree with and erased this extrapolated sentence from the manuscript. |
| **Background** |  |
| 1.The first sentence in the Background talks about when C-sections are justified or not. However, this is not discussed further in the text. A better topic statement regarding the premise of the study could be considered instead of starting with the claim whether C-sections are justified or not. | Thanks a lot for your comments. We have corrected the relevant part according to your suggestions. |
| 2.The authors state: “During the past 30 years there was an increasing trends of practice of C-section, it varies from 0.4 to 40 percent among countries around the world [1]”. However, the study they cite is from 2006 and is based on data from 1993 to 2003. A correct citation is required. | Thank you for your comments. Appropriate correction with proper citation has been done. |
| 3.The authors again state that: “In a recent study, which included data from 169 countries across the globe in 2015, the overall C-section rate was reported to be 21.1%, twice as high as that estimated based on 2000 [4]." However, the reference for this statement refers to a 2015 cross-sectional study in Tehran! | We corrected the mentioned information along with pertinent reference. Thanks a lot for your invaluable comments. |
| 4.“Previously, it is observed that economically developed countries have a higher rate of C-section.” This requires a reference. | Thanks a lot for your precious comments. Suitable references has been added. |
| 5.“The developed countries are strictly imposing restrictions on unnecessary C-sections.” – This requires a reference. | Thank you very much. Proper reference has been included. |
| 6.“There is a range of 0 to 10 percent of C-section among 76 percent of low-income countries, 16 percent of medium-income countries, and 3 percent of high-income countries. In addition, the distribution of having more than 20 percent of C-section is as: three percent in the low-income countries, 36 percent in medium income countries, and 31 percent in high-income countries [1].” The figures are again derived from a study performed on the data from 1991 – 2003. The authors need to report recent figures and provide the information in concise manner. The given text is difficult for a reader to follow. | Arranged accordingly. |
| 7. The discussion pertaining to risks of C-sections is incoherent, lacks appropriate references (only studies from early 2000s are cited) and requires more work to be articulate. For example, the authors mention injury and disability as a risk of C-section. However, it is unclear from the text who is at risk, the mother or the baby or both. | Thanks a lot for your comments. We tried to include literatures that are more recent as reference. We now clearly mentioned that both mother and baby is at risk of injury and disability. |
| 8.The authors should note the revised statement regarding the recommended rate of C-sections (the commonly cited 10-15% range): “At population level, caesarean section rates higher than 10% are not associated with reductions in maternal and newborn mortality rates.” [link: <https://www.who.int/publications/i/item/WHO-RHR-15.02>] | Thank you for your concern. We have included the updated information at the pertinent place. |
| 9.Please fix the idiosyncrasies in the text: According to the WHO report 2010, there were 6 million elective C-section practiced performed in 2008 and caused cost which resulted in an estimated cost of….”. There are more of such inconsistencies. | Correction has been made. We cordially appreciate your comments. |
| 10.“C-section in the private hospitals usually summed up higher medical care cost compared to government hospitals” – Please provide a reference from the countries discussed in the paper. | Thanks a lot for your comment. We included proper reference according to your suggestion. |
| 11.The authors state that, “Literatures found a number of determinants which are associated with the implementation of C-section including maternal age, maternal weight, parity, prolonged labour, mother with HIV infection, previous C-section delivery, dystocia, breech presentation, placenta previa, and suspected fatal complications [22-25]” – | Thanks for arising the issue; we have corrected the mentioned information with suitable recent citations. |
| a.The authors need to provide information on the class of determinants the existing literature discusses, rather than listing them as is currently provided. | Thanks again for your valued comment. We have tried to figure out class of determinants as you suggested. |
| b.The references here are from 1996, 1999, 2006 and 2007. The authors need to include up to date references. There are other important studies the authors failed to discuss in the literature review. For example, the study claims that one of the main motivation behind the research is the rise in elective C-sections but fails to discuss the following papers that analyses the determinants of Elective C-sections in Bangladesh (<https://doi.org/10.1038/s41598-021-96337-0>) and another relevant paper (<https://doi.org/10.3390/ijerph19031465>). | Many thanks for your comments and suggestions. We have updated references by including more contemporary and relevant ones. We also cite the your suggested papers. |
| **Methods** |  |
| 1.The use of the phrase “waves of DHS” in this context is imprecise, please use the phrase "round of DHS". Also, the authors should refrain from using terms such as “cherished objectives”. | We warmly implemented the corrections as you suggested. Many thanks for your suggestions. |
| 2.Section 2.1 is poorly worded. A brief discussion on study design should be included. | Thanks a lot for your comment. We expanded section 2.1 as you suggested. |
| 3.The references 30-32 are completely unnecessary (they are unrelated papers, and all are based on Bangladesh) and should be replaced with the DHS report books for each country. | Thank you very much. References has been updated by replacing with the most recent DHS reports of each country |
| 4.The authors need to clarify what they mean by “Only the institutional deliveries held on governmental or non-governmental healthcare centres are considered as C-section deliveries.” Does this mean all institutional deliveries are C-sections? Which would not make sense. | We appreciate your concern. We mean deliveries held at home cannot be C-section. Chunks of institutional deliveries are C-section. |
| 5.“parity, number of ANC visits” need to be clearly defined. The acronym ANC does not have the full form. | Thank you very much. We have done the suggested corrections. |
| 6.“For model selection, backword elimination procedure was followed.” The model selection should be based on sociodemographic domain and theoretical considerations. | Thanks again for your comments. Exposure were included in the study based on sociodemographic domain and theoretical point of views. However, for the final regression model selection we applied backward elimination procedure. That means we run the model including all the exposures and eliminate systematically based on the statistical significance. |
| 7. Instead of latest versions, please provide the version number for the statistical software used. | Statistical software version has been mentioned accordingly. |
| Results |  |
| 1. Information in tables 1 and 2 could be more concisely presented in a line graph with appropriate labelling, showing the trends for each country. | Thanks for your suggestion. We have added line graphs showing the trends of birth at hospital and C-section for the three countries: Bangladesh, Nepal, Pakistan. |
| 2. The authors should make sure they use the words enhanced and increased in a consistent manner. | Thanks a lot for your comment. We affectionately accepted your suggestions. |
| 3. The study is cross-section in nature; however, the authors use causal language to describe the associations. For example, the authors wrote, “For every one-year increase of women’s age, the risk of caesarean section is approximately 1.07, 1.11 and 1.02 times in women as compared to women with normal delivery in Bangladesh, Nepal and Pakistan respectively”. The correct form of interpretation should be, “For women who differ in age by 1 year but possess the same levels of other variables, the odds of having a C-section birth is approximately 1.07, 1.11 and 1.02 times the odds of having a normal/vaginal delivery in women from Bangladesh, Nepal and Pakistan respectively.” | Many thanks for your comments. We lovingly accepted your suggestions and did the modifications in the relevant part. |
| 4. Similar misinterpretation of Odds Ratio statistics can be seen in the form of “2.52 and 2.64 times more likely underwent caesarean delivery”. Likelihood is not equivalent to Odds ratios. | Thanks a lot for the comment. We do appreciate your suggestions and have implemented the modifications. |
| **Discussion** |  |
| 1. The manuscript says, “Along with the increase of delivery at facility the rate of C-section increases rapidly echoes unnecessary use of healthcare facilities” – This is a very strong statement to make from an observational study and without proper references. Furthermore, the response variable was a binary variable for having C-section or not. There is no further evidence from the study to support that most of the increase are due to “unnecessary use of healthcare facilities”. The reference cited (WHO study from 2006) only speaks to the second part of the claim “…. detrimental future effects for both mother and kids”. | Thank you very much for your comments. We have changed the sentences. |
| 2. “In Bangladesh the prevalence of C-section were more than two times in 2017” – Unclear statement. Two times regarding what year is unclear. | Thanks again for your concern. We have corrected the statement. |
| 3. “Other studies findings also bolster our assertion [37, 38].” – This is redundant as both these studies use the DHS surveys used in the current manuscript and are of similar analyses. | We sincerely accept your comment. We have erased this redundant statement. |
| 4. “What are the reasons behind of such skyrocketing rates of C-section? Is only the improvement of medical science and healthcare facilities being the main cause?” - This is a very minor issue, but authors should refrain from asking direct questions to the readers, but rather pose them study inquiries. | Thanks for raising this important point. We have changed accordingly. |
| 5. “Most of the unnecessary C-section occurred due to mother’s misconception of delivery pain, convenience and lack of patience [43, 44]” – the references are from 2001 and 2003 studies based in the UK, which is demographically quite different from the countries discussed in the paper. The authors must remove these and include relevant and contemporary studies. | You have raised an important point. We have already updated the mentioned references by contemporary and relevant studies. |
| 6.“Besides, physicians intention of earning more money is another significant factors of worldwide upsurge of elective C-section [45, 46]” – This again a very strong claim to make using references from 2003 and 1993, and may be outside the scope of the performed analyses without clarifying how the physicians stand to make more money since it is not mentioned whether physicians in Govt facilities in these countries are paid a specified salary or a variable salary based on the number of C-sections they perform. | Thank you very much for mentioning this point. We have changed the references and placed recent and pertinent studies to support the claim. |
| 7.“This association resonant with other studies [36, 38, 47, 48].” This is again a redundant claim since the first two studies used the exact same data (except maybe missing the most recent DHS). | We have deleted the redundant references according to your suggestions. Thank you for pointing out this issue. |
| 8.“Premature and older women were adversely associated with reproductive and pregnancy related risks. Several studies divulged that elderly pregnant women underwent highly risks of preterm delivery, low birth weight, perinatal death, and CS [49, 50].” – Unclear context. | Thank you very much. We have completed the corrections. |
| 9.“In Bangladesh and Nepal urban women are more inclined to exploiting C-section, but in Pakistan rural women are more prone to C-section.” – Not sure how the authors came to such inference from statistically non-significant results (Table 4). | We are so sorry for the unintentional mistake. Thanks a lot for your comment. We have corrected this statement. |
| 10. “increased BMI because of westernized food cultures may be the possible reasons of increased use of C-section” – This may be over-interpreting the scope of the analyses performed in the manuscript. BMI increase could be due to other factors such as post-partum issues. There is no indication in the data that the BMI is pre-pregnancy BMI. | Thank you very much for your concern. We have amended it, though we have not claimed it. Just said it may be a possible reasons. |
| 11.“In Bangladesh and Pakistan, the likelihood of choosing C-section by pregnant women were associated with the educational attainment of both women and their husband matching with the results of other studies [27, 35, 36, 47].” - The authors cannot repeatedly claim other studies support their findings if those studies used the exact same data and analysis methods. For example, “In case of Nepal women education was not associated with the C-section rates, which contradicts with the previous studies [37, 38]” – this is an interesting result which requires more discussion with respect to the sociodemographic differences in Nepal compared to Bangladesh and Pakistan. | Thanks again. We tried to correctly modify the mentioned parts according to your suggestions. |
| 12.“… Similar pattern was documented in the previous studies [34, 36, 38, 48]” – Similar issue. | Thanks a lot. The issue is similar in the mentioned studies but different rounds of DHS and for individual countries were explored. |
| 13.“Mothers during their second, third or more order of birth giving may be less intimidate the pain of labour as well as they develop the physical and mental ability to cope with the hazards of C-section.” – This is confusing. Are experienced mothers less intimidated by vaginal birth or C-sections? The study found age was positively associated with higher odds of C-section. | Thank you very much. The statement is not related to age, it is connected with the birth order. The more the birth order the less the odds of C-section. |
| 14.“Maybe more number of ANC influence anxious mothers to choose the C-section, or physicians inspire them to undertook C-section for getting more incentive from the hospitals.” – These claims need to be supported by evidence. | We appreciate your comment. Proper citations for the statement has been provided. |
| 15.“It is expected that within 2026 Bangladesh will enter to the elite class of middle-income countries.” – The authors should provide references to such over-optimistic claims. | Thank you very much. We have added reference in favour of the statement. |
| 16.“As socioeconomic conditions have improved the ability of bearing healthcare costs of peoples also increased, that is why people prefer to go to the private hospitals for getting more comfortable healthcare than the public hospitals.” – Attributing people’s health seeking tendencies to economic development only is problematic. This again goes beyond the scope of the paper. | We appreciate your comment and we have tried to modify this statement. |
| 17.“Several prior studies in different developing countries in the world also exhibited the comparable and consistent results to our findings [27, 34, 55-59].” References 27, 34, 55 etc. are studies based in Bangladesh and Pakistan using same data. Please remove them. | Thanks a lot for your suggestion. We have removed the mentioned citations. |
| **Conclusion** |  |
| 1.“As policy implications we are suggesting social media awareness for reducing unnecessary C-section.” This is the final concluding remark. However, the authors never really discussed why such methods would work in the context of this specific public health problem. | Thank you very much for your comment. Yes, you are completely right; it is beyond of our study. We have removed this statement. |
