# Supplementary material for: Trends and determinants of caesarean section in South Asian countries: Bangladesh, Nepal, and Pakistan
Source: PLoS One. 2024 Dec 5;19(12):e0311082. doi: 10.1371/journal.pone.0311082 (PMC11620693; doi:10.1371/journal.pone.0311082)
Supplement: Attachment — Submitted filename: Response to Reviewers_2nd.docx [file pone.0311082.s003.docx]

We appreciate your time in reviewing this manuscript and providing quality feedback. Our responses to your specific comments are added below.

| **Reviewer 2** | **Author responses** |
| --- | --- |
| 1. The authors still claim in the abstract and in the manuscript that “Through modification of influential factors (body mass index, birth order, wealth, and education) respective authorities should minimize the over reliance on C-section during childbirth.” - For example, how can “authorities” influence “birth order”, given that the authors show in their analyses that “there is an 88% less chance of caesarean delivery among the women who are giving more than four birth than the women who are giving the first birth)”? Do they mean they want the authorities to make sure that women avoid their first few births to lessen the odds of a C-section? How does that even make sense? Or do they want only older women with many children to give birth in the future? This may sound harsh but the authors, I think, do not really think about the implications of their recommendations. What they can really argue to be at play is that younger mothers tend to show a tendency to opt for a C-section, which may be portrayed to them as an easier/lower-risk/lower-painful option under anaesthesia compared to normal vaginal birth. This is just one example of incoherent discussion and interpretation by the authors. - Again, how can modifying education level help reduce C-section? The authors show in their analyses that higher the education level, the higher odds of observing C-section. Do they mean they would like the authority to curtail women’s education to keep C-section rates low? This does Not make sense. | Thanks for your detailed comments with examples. We agree with your comments and have made a substantial revision of the whole manuscript, including the abstract, results and discussion sections. The mentioned contradictory statements have been deleted, and appropriate interpretation has been added accordingly. For example, the conclusion of the abstract has been revised as  *“Our findings regarding the association of sociodemographic factors with increased C-sections may help identify subgroups of women susceptible to C-sections and offer better support regarding C-sections plans. However, the substantial increase in C-sections across the three countries warrants further investigation to identify the reasons.”*  The abstract has also been reduced to 298 words compared to 400 words in the previous version (the journal requirement is 300 words).  According to your comments, we have revised all the contradictory interpretations throughout the manuscript, especially in the "Results" and discussion sections. We believe the current manuscript has been substantially improved by addressing your insightful comments.  Please see all changes in the track-change version of the manuscript. |
| 2. The authors finally argue their rationale for selecting the 3 countries that they chose based on the fact that “they have several similarities in terms of maternal and child health: high maternal and child mortality rates, limited access to quality health care, malnutrition and undernutrition, maternal health services, socio-economic and cultural factors” as well as similar challenges. However, at the same time they highlight that Nepal is different from the other two countries later in the paper and this is also apparent from their results. Furthermore, they do not provide any evidence/reference to the reader for the above argument for their study rationale. | Thanks for this comment. We have revised the text in the last paragraph of the Introduction section as follows, along with four references (Akseer et al., 2017, Bhutta et al., 2004, Anik et al., 2019, and Misu & Alam, 2023).  “…*These three countries faces similar challenges in maternal and child health issues [29, 30]. Despite some differences in specific indicators and methods, they possess similarities in terms of maternal and child health: high maternal and child mortality rates, limited access to quality health care, malnutrition, maternal health services, and socio-economic and cultural factors [31, 32].* *Moreover, all three countries face challenges regarding healthcare infrastructure, skilled healthcare professionals, and availability of essential drugs and equipment. Thus, this study focused on these three South Asian countries to determine and compare …*”  Please see the changes in detail in the track change version of the manuscript. |
| 3. In response to the comment “The author must include the variable "type of hospital/facility" in which Caesarean Section delivery have been performed in this analysis. It helps to understand why CS rises, especially because of private facilities across the counties”, the authors do not acknowledge its importance and shy away by saying that they will incorporate this into their future study. Which makes no sense as whether the current manuscript will be judged on what is included in this one and not what the authors promise to do for their next study. | Thanks for your comment. In every relevant case, we have considered the suggested variable, re-analysed the data, and modified the results accordingly. For instance, we added “*place* *birth (private and public hospitals)*” in the list of explanatory variables in subsection 2.3. We summarized the newly fitted logistic regression results in Table 4, along with adjusted odds ratios (AORs), confidence intervals, and p-values. We added the following text in the last line of the Results section.  “*The significantly increasing odds of C-sections in private hospitals compared to the public hospitals were observed in all three countries (Bangladesh: AOR = 5.31, CI = 4.76, 5.93; Nepal: AOR = 2.03, CI = 1.61, 2.56); Pakistan: AOR = 1.30, CI = 1.08, 1.54)*.” |
| 4. One of the main arguments of their study is the “WHO recommended standard level of C-section (10%-15%)”. However, this figure has been widely criticized and even the WHO no longer supports this figure in the context of the current scenario across the world. | We sincerely agree with this comment. As we do not have any other standard reference value, we added the following line in the Discussion section with a reference by Betran et al. and the WHO Working Group on Caesarean Section, 2016.  “…*Although there is a controversy and there is no international consensus regarding this rate, we used it for baseline comparison…*”  We also added a similar text in the Introduction section as follows (2^nd^ paragraph 2^nd^ line from bottom).  “…*Although there is a controversy and there is no international consensus regarding this rate, we still kept this number for baseline comparison*…” |
| 5. The authors continue to use inconsistent and irrelevant references to support strong claims. One example is: “Besides, physician’s intention of earning more money is another significant factors of worldwide upsurge of elective C-section [39]” This reference comes from a Swedish study, which is not relevant to LMICs. Even the authors do not argue how a study on economics of C-section in a developed country can be used to justify a claim in the context of the entire world. | Thanks for your comments. We have updated the text with relevant reference (Lambert, 2020) as follows.  “*Besides, relative underpayment of physicians across the three countries, many intend to have inappropriate financial benefits, which may also result an upsurge in elective C-sections [46]*” |
| 6. The study still misses the mark of its intended target. For example, “Our study also depicted that there is inequality of the prevalence of C-section by place of residence of the mothers. In Bangladesh and Nepal, urban women are more inclined to undergo C-section, but in Pakistan, urban women are less prone to C-section.” The authors do not go into discussing why this might be case or why the difference exists for rural women of these three countries. They provide convoluted sentence a bit later trying to apparently justify all the interesting results at once, which again makes little sense. The paper is about understanding the dynamics, similarities, and dissimilarities of the three discussed countries that “share a lot of the same demographic characteristics”. | Thanks for this constructive comment. It is mentioned earlier in response to the reviewer’s first comment that the Discussion section has been revised substantially with relevant and newly added references. For instance, the following text has been added in the Discussion section to explain the possible reasons for differences in the C-section rates between rural and urban areas.  “…*We also observed a significant difference in the prevalence of C-sections by mothers’ place of residence. In Bangladesh and Nepal, urban women are more inclined to undergo C-sections, but in Pakistan, urban women are less prone to C-sections. Previous researchers also found similar results [41, 47, 52, 53]. The reasons of lower rate of C-sections in rural areas in Bangladesh and Nepal could be due to a number of factors such as lack of accessibility to hospitals, customs and traditions of the society, financial ability, awareness, and others [54-58]. Availability of improved medical facilities, more antenatal care (ANC) visits, the tendency to take children at a later age, high socioeconomic status, highly educated mothers and their partners, increased BMI, and post-partum reasons may be some possible reasons for increased use of C-section in the urban regions. However, the lower number of C-sections among urban women in Pakistan warrants further investigation...*”  We have not listed every change and addition here. We refer to the track change version of the manuscript to see all changes made according to the reviewer’s comments. |
| 7. The authors made little attempt at fixing the Discussion section with one or two minor changes, which was one of the most problematic areas of the original manuscript. | Thanks for your constructive comments. In response to the reviewer’s comments 1 and 6, we tried to explain how extensively we revised the Discussion section (along with other sections) with some newly added relevant references. To avoid repetition, we are not adding any "text" examples here. We want to refer to the track-change version of the manuscript to see all changes. |
| 8. The strengths and limitations discussed are also very generic and do not speak to the issues unique to this study. | Thanks for raising this important issue. We have changed this section to mention important strengths and limitations. Please see the changes in the track-change version of the manuscript. |
| 9. The manuscript still offers very little insight into the possible dynamics of the trends and determinants, which was their main goal of the study. | We appreciate your comments regarding this. We have presented the trends in Tables 1 and 2 in the manuscript and Figures A1 and A2 in the appendix. We have also added p-values obtained from the Cochran-Armitage test to assess the statistical significance of the reported trends. To identify the significant determinants associated with the C-section, we performed the bivariate analysis with chi-square tests and fitted multivariable logistic regression for covariate adjustment. The results are summarized in Tables 3 and 4, respectively. |
| Minor points |  |
| There are still quite a few grammatical issues and these need to be fixed. | Thanks for checking this. We carefully reviewed the whole manuscript and corrected all the grammatical mistakes. Moreover, we substantially modified the language throughout the manuscript (please see changes in the track-change version of the manuscript). |
| If possible, the authors can also consult a public healthcare/medical professional regarding the findings of their analyses to put their results into more context so that it can be helpful to policy discussions in the future. | Thanks for your suggestions. We revised the whole manuscript with the help of public health experts., including Dr. Khan and Dr Rahman, who are currently the new coauthors, given their intellectual contribution. |
