# Supplementary material for: Trends and determinants of caesarean section in South Asian countries: Bangladesh, Nepal, and Pakistan
Source: PLoS One. 2024 Dec 5;19(12):e0311082. doi: 10.1371/journal.pone.0311082 (PMC11620693; doi:10.1371/journal.pone.0311082)
Supplement: Attachment — Submitted filename: Response to Reviewers.docx [file pone.0311082.s004.docx]

We appreciate your time in reviewing this manuscript and providing quality feedback. Our responses to your specific comments are added below.

| **Reviewer 1** | **Author responses** |
| --- | --- |
| The author has revised the manuscript, making it more presentable. The findings of the study will highlight the progress of cesarean births in South Asian countries. | Thanks for your valuable comments throughout the review process which improved the quality of the manuscript. |
| **Reviewer 2** |  |
| The current version of the manuscript is substantially improved and well written. I thank the authors for their patience and effort to address all my concerns. I believe the manuscript can now be accepted in its current form after the following minor comments have been addressed. | We appreciate your precious inputs and constructive criticisms. Your comments and suggestions help us a lots in improving the quality of the paper. |
| 1. The figures A1 and A2 should be fixed in the following manner:  a. Both figures should the same same X-axis. A1 has x = 0, while A2 does not. Furthermore, the axis ticks and marks also differ for the two plots. They should be the same and have the same starting point.  b. There should be uniform color for the same country in the two plots. For example, Bangladesh has blue and black trendlines in the two plots. It should be the same color. The color scheme for Nepal is a good example.  2. The keyword "Cesarean" in the submission portal information (first page of the generated pdf) spells differently from what is followed in the manuscript (Caesarean). Please take note and maybe fix it in the final version. | 1. Thanks a lot for pointing out the issue. We fixed it; please have a look at the track change version of the manuscript.  2. We changed all the “cesarean section” with “caesarean section” on the revised manuscript. |
